# Vascular Compression of the Esophagus: A Case of Dysphagia Aortica

**DOI:** 10.7759/cureus.106727

**Published:** 2026-04-09

**Authors:** Arman Manjikian, Michael G Ghobrial, Victoria Diaz, Tarek Ammar

**Affiliations:** 1 Internal Medicine, MountainView Hospital, Las Vegas, USA; 2 Internal Medicine, Sunrise Health Graduate Medical Education Consortium, Las Vegas, USA; 3 Gastroenterology, Comprehensive Digestive Institute of Nevada, Las Vegas, USA

**Keywords:** barium esophagram, ct angiography, dysphagia aortica, elderly dysphagia, extrinsic esophageal compression, pseudoachalasia, thoracic aortic aneurysm, vascular dysphagia

## Abstract

Dysphagia aortica is a rare cause of external esophageal compression from an enlarged, tortuous, or aneurysmal aorta. An 85-year-old woman with a known ascending aortic aneurysm presented with several weeks of intermittent regurgitation of liquids and pills. A barium esophagram revealed a severe 40 mm long segment narrowing of the distal esophagus with delayed transit. Computed tomography angiography (CTA) revealed a 5.6 cm ascending aortic aneurysm exerting mass effect on the esophagus; endoscopy noted no intrinsic obstruction or obvious external compression. This case demonstrates the challenge of diagnosing dysphagia aortica in the setting of normal endoscopy, emphasizing the need for additional imaging.

## Introduction

Dysphagia aortica is an infrequently diagnosed condition characterized by esophageal obstruction from extrinsic compression from an enlarged, tortuous, or aneurysmal thoracic aorta. First described by Pape in 1932 [1], it primarily affects older adults with long-standing hypertension or degenerative aortic disease. Less than 100 cases have been described in published literature [2]. Diagnosis is often delayed because endoscopic findings are frequently normal [3], emphasizing the importance of appropriate radiographic evaluation. Here, we present an 85-year-old woman with a known history of an ascending aortic aneurysm who presented with several weeks of progressive dysphagia and was found to have significant esophageal narrowing despite normal endoscopic findings.

## Case presentation

**Figure 1:**
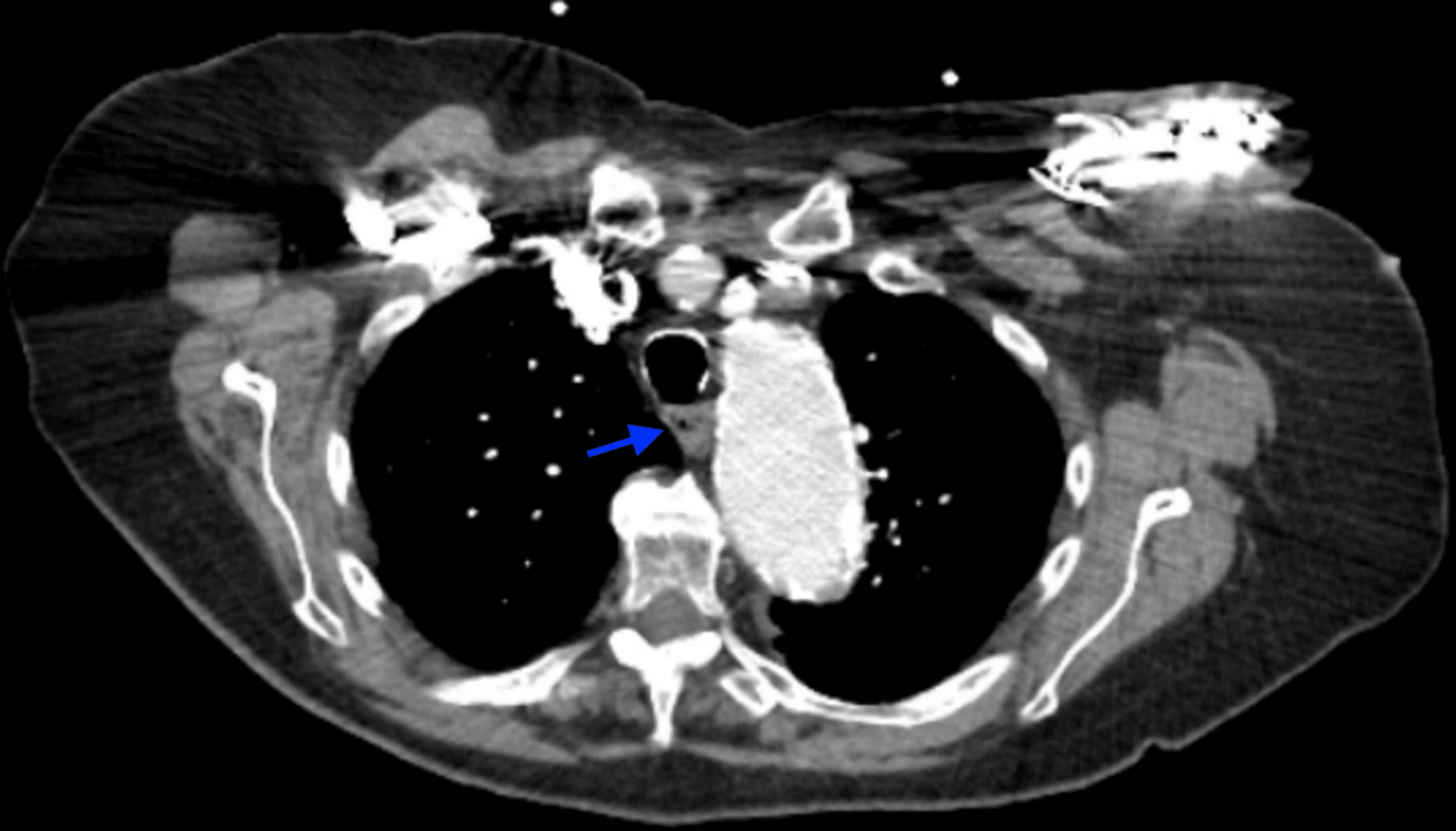
CTA in axial view The blue arrow points to a compressed esophagus by an aortic aneurysm. CTA: computed tomography angiography

**Figure 2:**
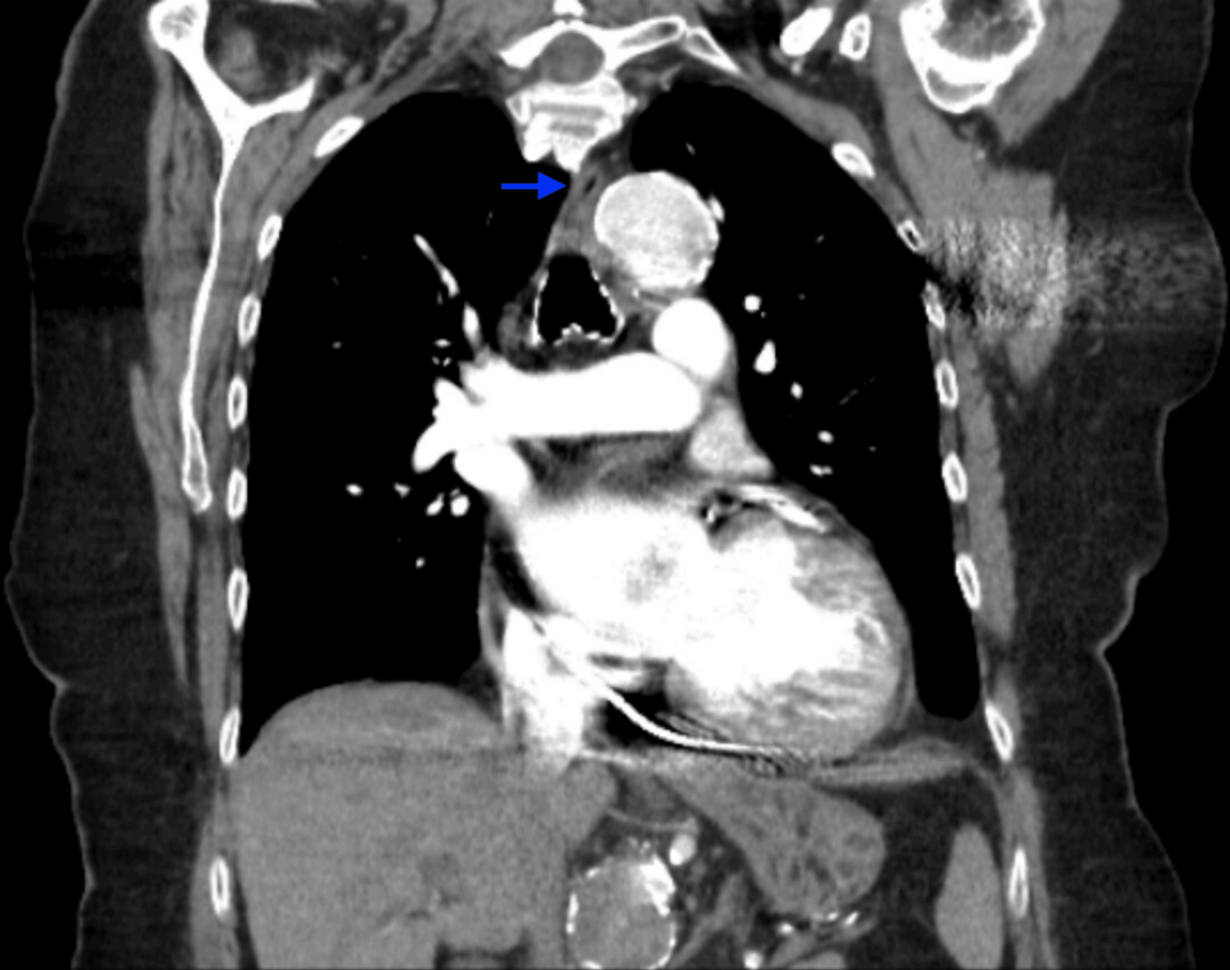
CTA in coronal view The blue arrow points to a compressed esophagus by an aortic aneurysm. CTA: computed tomography angiography

An 85-year-old woman with a known ascending aortic aneurysm presented with several weeks of intermittent regurgitation of liquids and pills. A barium esophagram revealed a severe 40 mm long segment of narrowing in the distal third of the esophagus with delayed passage of contrast into the stomach. Physical examination was unremarkable. Computed tomography angiography (CTA) of the chest revealed a 5.6 cm ascending thoracic aortic aneurysm exerting mass effect on the esophagus (Figures 1, 2). A modified barium swallow did not show aspiration but reproduced regurgitation of contrast material. Upper endoscopy revealed a normal esophagus with no mechanical or intrinsic obstruction appreciated. Overall findings favored extrinsic compression from the aortic aneurysm as the etiology of her dysphagia.

Initially, diet was held, but she was later able to tolerate small sips of liquid diet under speech therapy guidance. Nephrology was consulted and managed hydration needs and provided parenteral nutrition because of her limited oral intake. Cardiothoracic surgery was consulted during this admission, but the patient declined operative repair. A feeding tube was offered, which she also declined. Palliative care was consulted, and the patient opted for conservative management focusing on comfort. She was discharged home with home health services.

## Discussion

Dysphagia aortica is an uncommon cause of esophageal obstruction in older adults, usually with chronic hypertension, atherosclerosis, or age-related aortic elongation [1,2]. Symptoms arise from progressive dilation of the thoracic aorta exceeding 5-6 cm, leading to mechanical compression of the esophagus. Our patient developed symptoms at a thoracic aortic diameter of 5.6 cm, consistent with the symptomatic range [4,5].

The misleading feature of dysphagia aortica is unremarkable endoscopic anatomy despite abnormal imaging findings. Endoscopy typically shows normal-appearing mucosa without obvious extrinsic luminal compression, which can misguide diagnoses toward functional or reflux-related etiologies [3]. In contrast, barium esophagram and CTA typically show the underlying causal mechanisms, which include delayed transit, posterior indentation, or direct mass effect on the esophagus [2,4]. This incongruity was evident in our case, where normal endoscopic evaluation markedly differed from the severe narrowing seen on imaging.

Management differs significantly between cases where patients pursue operative repair and those who decline intervention. Surgical or endovascular treatment can relieve symptoms and address aneurysm-related rupture risk [4]. However, many elderly or medically complex patients decline surgery due to operative risk or personal preference. In such cases, as in our patient, management focuses on diet modification, speech therapy strategies, hydration, and nutritional support. These conservative measures may provide partial relief but do not correct the underlying mechanical obstruction.

## Conclusions

Dysphagia aortica is an uncommon but important cause of esophageal obstruction, particularly in older adults with aneurysmal or degenerative aortic disease. This case highlights how significant aortic compression may produce symptoms despite a normal endoscopic evaluation, emphasizing the need for radiographic imaging when dysphagia remains unexplained. Early recognition is important to avoid delays in diagnosis and prevent symptoms from being attributed to more common esophageal conditions. Clinicians should keep this diagnosis in mind in patients with known thoracic aortic dilation who develop persistent dysphagia despite normal endoscopic findings. Timely use of imaging studies, such as barium esophagram and computed tomography angiography, can help confirm the diagnosis and guide appropriate management decisions.
